# Cancer incidence in an Austrian alpine valley 1983–2012: A descriptive study

**DOI:** 10.1007/s00508-019-1476-7

**Published:** 2019-03-14

**Authors:** Hans-Peter Hutter, Thomas Waldhoer, Katja Müller, Monika Hackl, Lisbeth Weitensfelder, Harald Heinzl

**Affiliations:** 1 Department of Environmental Health, Center for Public Health, Medical University of Vienna, Vienna, Austria; 2 Department of Epidemiology, Center for Public Health, Medical University of Vienna, Kinderspitalgasse 15, 1090 Vienna, Austria; 3 Austrian National Cancer Registry, Directorate Social Statistics, Statistics Austria, Guglgasse, Wien, Austria; 4 Section for Clinical Biometrics, Center for Medical Statistics, Informatics and Intelligent Systems, Medical University Vienna, Vienna, Austria

**Keywords:** Epidemiological study, Cancer incidence, Görtschitztal valley, Hexachlorobenzene, Mesothelioma

## Abstract

After one of Austria’s largest environmental scandals in 2014, which involved the release of hexachlorobenzene (HCB) in the Carinthian valley Görtschitztal, concerns about increased cancer rates have arísen in the affected local population. A descriptive study was conducted to examine the cancer incidence rates between 1983 and 2012. Data from the affected area (Görtschitztal, district St. Veit) were compared to data from the neighboring area within the same district and Carinthia excluding St. Veit, considering incidence rates of liver, lung, kidney, thyroid cancer and mesothelioma. Prostate cancer and carcinoma in situ were both included and excluded from overall cancer incidents in order to prevent potential bias due to screening programs. Considering the observed variability at an overall level, no conspicuous differences in cancer incidences could be found (Carinthia: 495, St. Veit West: 408, St. Veit East: 572 cases per 100,000 person-years in 2012). For some cancer types, e. g. liver, thyroid cancer and mesothelioma, the affected region showed a higher increase in rates than the neighboring area or Carinthia overall; however, these increased rates date back to a time prior to the HCB exposure, suggesting other carcinogenic influences, such as asbestos exposure from antecedent years.

## Introduction

The hexachlorobenzene (HCB) contamination in the Carinthian valley Görtschitztal (southern Austria) was one of the largest environmental scandals in the recent history of Austria. During routine testing of a bio-ricotta cheese from a local producer in this region, HCB was detected early in March 2014. The hexachlorobenzene was identified in other food products as well as a result of contaminated animal feed and contamination of soil and plants. Although the concentration did not exceed the level of the European Union pesticide residues directive (EC 396/2005) the authorities were notified and a search for the origin of HCB was initiated. During November 2014 legal limits for milk products were exceeded for the first time as well as values in animal feed.

In the Görtschitztal valley two lime disposal sites are located, which are owned by a chemical factory. Between 1926 and 1981 these landfills were also used for deposition of hazardous production residues with substantial amounts of chlorinated hydrocarbons, among them also HCB. The deposit was classified into the highest priority class for remediation by the Austrian Federal Environmental Agency in 2003 [1]. As a consequence, the chemical factory was obliged to perform or commission remediation procedures. In 2011, a nearby cement factory was licensed for incineration of the contaminated sludge under certain restrictions. In order to degrade HCB, high temperatures are required (>800 °C). The cement factory located approximately 12 km from the deposit claimed to be able to adhere to these requirements. An intensified search for potential sources of the contaminated dairy products established in November 2014 revealed that due to insufficient temperature during combustion of the contaminated lime sludge, HCB was released into the air for an unknown duration but likely since mid-2013. Subsequently, incineration of slaked lime was prohibited by the authorities and a warning was issued for consumption of agricultural products from the Görtschitztal region. This caused significant distress to the region’s population where farming and in particular organic farming is a significant source of income. At this time of great uncertainty, discomfiture and distress for the public, a newspaper article claimed “higher cancer incidence in the HCB district for the time period 2009–2011” [2]. This article also included a choropleth map illustrating higher cancer incidence rates in Carinthia, and particularly within the district St. Veit/Glan. As a result, widespread concerns about potentially elevated cancer risks emerged in the Görtschitztal region which also includes the eastern region of the district St. Veit/Glan.

The effects of environmental contaminants on cancer incidence may be identified only after long latency periods. Since the increased exposure to HCB occurred after the time period covered by the reported incidence data, an effect of the release of HCB on cancer incidence can be ruled out, but it still raised substantial concerns among the public. Therefore, a detailed descriptive epidemiological analysis was initiated aiming to identify whether the incidence of cancer within the Görtschitztal region was increased compared to the rest of Carinthia [3].

This study aimed to clarify a) whether overall incidence rates of different types of cancer in Görtschitztal valley changed during the years 1983–2012, b) whether specific incidence rates of liver, lung, kidney, thyroid, and mesothelioma cancer changed in this time period and c) whether there are differences in cancer incidence rates between Görtschitztal valley and the rest of Carinthia. Cancer entities were based on toxicological considerations and selected by expert interviews including employees of local public health authorities.

## Material and methods

Incidence of overall cancer (defined as ICD-10 codes C00–C97 [excluding C44] and D00–D09 [excluding D04]) and specific cancer incidences in Görtschitztal valley in comparison to the rates in the region of Carinthia, between 1983 and 2012 were computed. The data used in this study were collated on 2 October 2015.

Cancer incidence data were obtained from Statistics Austria. No ethical approval was required because only aggregated data were used in such a way that identification of individuals was not possible. To ensure data protection, Statistics Austria requires that a minimum of four municipalities are combined. The study satisfied this requirement by analyzing districts or parts of districts which are all composed of more than four municipalities.

Incidence rates of prostate cancer (C61) as well as carcinoma in situ (D00–D09) may be affected by local variation in opportunistic screening programs and completeness of case ascertainment [4]. Therefore, overall cancer incidence was calculated including as well as excluding prostate cancer and carcinoma in situ. Due to a lag in the completeness of the registry [5] the study period ended at the end of 2012. For the purposes of this study, the region (district St. Veit/Glan) was divided into St. Veit East (11 communities) and St. Veit West (9 communities). St. Veit East is virtually equal to the Görtschitztal region, where the lime deposits and cement factory are situated, and is referred to as St. Veit East throughout this report.

### Statistical analysis

Direct age-standardized rates of all malignancies in St. Veit East, St. Veit West and Carinthia including St. Veit/Glan as well as Carinthia excluding St. Veit/Glan were calculated. For tumor-specific analysis, incidence rates were combined into 5‑year intervals because of the low incidence of some cancers. Direct age-standardized rates and corresponding 95% confidence intervals (CI) were calculated in SAS 9.4 (SAS Institute Inc., Cary, NC, USA) using the European standard population (Annex F, p. 121 Revision of the European Standard Population Report of Eurostat’s task force, 2013 edition). Rates are expressed as cases per 100,000 person-years. Due to the lack of a priori hypotheses, no statistical tests for time trends or comparing incidence rates between regions were performed.

## Results

### Incidence of all malignancies

**Fig. 1:**
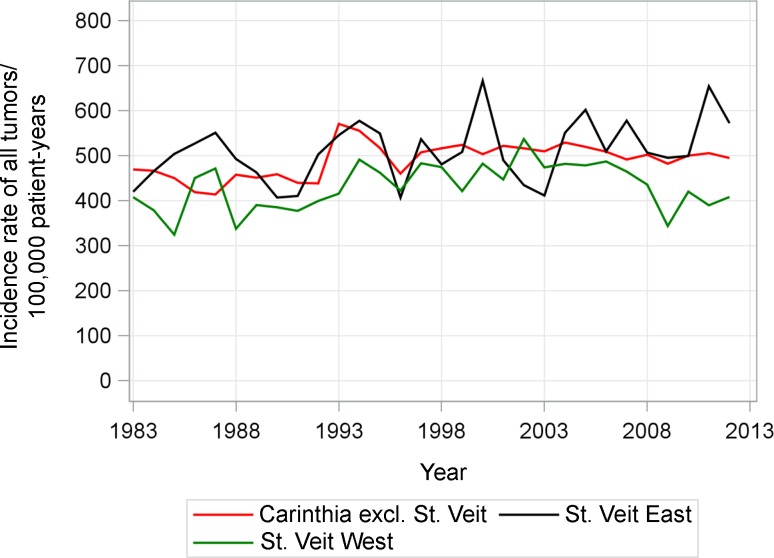
Age-standardized incidence rates of all tumor sites (ICD-10 C00–C97, excluding D00–D09, C44, C61) per 100,000 person-years in 1983–2012 (EU standard population, 2013)

In all districts of Carinthia, the incidence of cancer excluding prostate cancer and cancer in situ cases increased in the time period from 1983 to 2012. In St. Veit East the incidence of cancer was higher than in St. Veit West in 28 out of 30 years and higher than in Carinthia (23 out of 30 years) (Fig. 1; Table 1). For males, St. Veit East had higher incidences than St. Veit West (26/30 years) and Carinthia (24/30 years). For females, the incidences of all malignancies were greater in St. Veit East than in St. Veit West (25/30 years) and similar to the average for Carinthia (St. Veit East greater than Carinthia in 14 years).

**Table 1:** Age-standardized incidence rates of all tumor sites (ICD-10 C00–C97, excluding D00–D09, C44, C61) per 100,000 person years in 1983–2012 (EU standard population, 2013)

Year	Carinthia excl. St. Veit	St. Veit East	St. Veit West
	Rate (95%CI) per 100,000 person-years		
1983	469 (446–494)	420 (347–505)	408 (306–537)
1984	467 (443–491)	466 (386–558)	379 (280–504)
1985	450 (428–473)	504 (426–594)	325 (228–449)
1986	419 (397–442)	527 (446–620)	451 (338–589)
1987	414 (393–436)	551 (463–651)	472 (352–618)
1988	458 (435–481)	493 (415–581)	338 (244–456)
1989	451 (429–474)	463 (390–547)	391 (295–509)
1990	459 (437–481)	407 (339–486)	385 (290–503)
1991	440 (418–462)	410 (343–489)	377 (284–493)
1992	438 (417–460)	503 (424–593)	399 (297–525)
1993	571 (547–595)	546 (464–637)	416 (311–544)
1994	555 (532–579)	577 (498–667)	491 (383–621)
1995	517 (495–540)	550 (472–636)	462 (353–594)
1996	460 (439–482)	407 (341–482)	422 (324–542)
1997	507 (486–530)	537 (460–623)	483 (377–610)
1998	517 (495–539)	481 (410–560)	475 (370–600)
1999	524 (502–546)	508 (435–590)	421 (328–534)
2000	503 (482–525)	666 (583–758)	482 (376–610)
2001	522 (501–544)	490 (419–570)	447 (346–569)
2002	516 (495–538)	435 (369–509)	537 (421–675)
2003	510 (489–531)	412 (347–485)	474 (373–595)
2004	529 (508–551)	551 (477–633)	482 (371–616)
2005	520 (499–541)	602 (525–687)	478 (379–597)
2006	509 (489–530)	510 (439–588)	487 (388–605)
2007	492 (472–512)	578 (502–662)	465 (368–579)
2008	502 (482–523)	507 (438–584)	436 (342–548)
2009	482 (463–502)	495 (426–572)	344 (264–441)
2010	500 (481–520)	499 (432–575)	420 (331–527)
2011	506 (486–526)	654 (576–740)	390 (302–496)
2012	495 (476–515)	572 (500–652)	408 (320–514)

*CI* confidence interval

Excluding in situ cases and excluding as well as including prostate cancer, in both scenarios the incidence in Carinthia and St. Veit East stayed the same, St. Veit West showing slightly lower incidence rates than the other regions.

### Tumor-specific malignancies

**Fig. 2:**
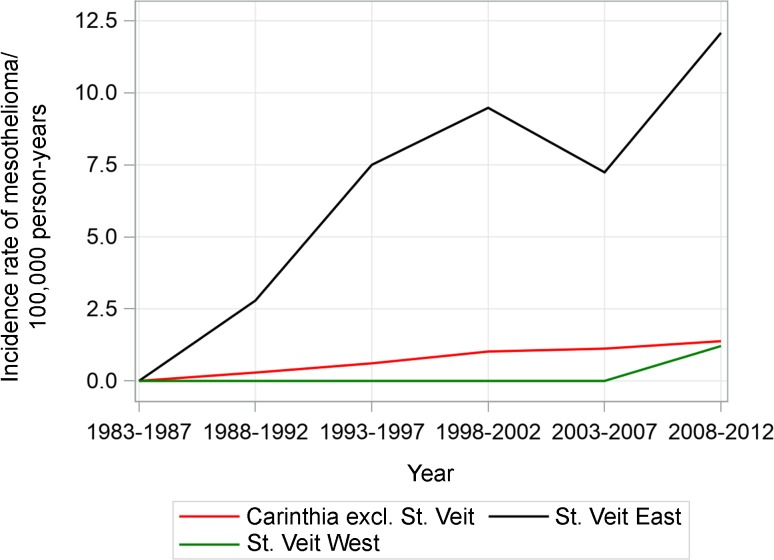
Age-standardized incidence rates of mesothelioma (ICD-10 C45) per 100,000 person-years, 1983–2012 (EU standard population, 2013)

For liver cancer, the incidence increased in St. Veit East, St. Veit West and Carinthia until 2008 and then decreased until 2012. St. Veit East had a higher incidence than St. Veit West and Carinthia for the majority of the studied period (5/6 of 5‑year intervals from 1983 to 2012). Lung cancer incidence was stable throughout the studied time period in all of Carinthia as well as St. Veit East. The incidence in St. Veit West decreased beginning with the period 1988–1992. Male mesothelioma incidence significantly increased in Austria overall during the studied period (from 2.9 to 4.4) [6]. In Carinthia, there was a slight increase in mesothelioma incidence rate (from 0 to 1.4) (Fig. 2; Table 2). St. Veit East had a higher incidence rate than St. Veit West throughout and increased steadily (2.8–12.1).

**Table 2:** Age-standardized incidence rates of mesothelioma (ICD-10 C45) per 100,000 person-years, 1983–2012 (EU-Standard population, 2013). No confidence limits are calculated for zero cases incidence

Year	Carinthia excl. St. Veit		St. Veit East		St. Veit West	
	Rate (95%CI) per 100,000 person-years, average population					
1983–1987	0	483,230	0	39,853	0	19,213
1988–1992	0.29 (0.10–0.66)	489,676	2.79 (0.90–7.35)	39,754	0	19,137
1993–1997	0.61 (0.33–1.06)	494,893	7.50 (3.97–13.07)	39,860	0	18,946
1998–2002	1.02 (0.64–1.56)	499,650	9.48 (5.36–15.50)	40,020	0	18,699
2003–2007	1.12 (0.73–1.64)	500,992	7.24 (3.93–12.24)	39,596	0	18,261
2008–2012	1.38 (0.95–1.93)	501,302	12.08 (7.63–18.18)	38,701	1.21 (0.03–6.74)	17,981

For kidney cancer, the incidence rates in St. Veit East and West were both above the average for Carinthia, with St. Veit West having a higher incidence rate than St. Veit East. Thyroid cancer incidence rate was higher in St. Veit East, compared to St. Veit West and Carinthia overall. Prostate cancer had a higher incidence rate in all of St. Veit compared to Carinthia overall.

## Discussion

An epidemiological study was undertaken in order to investigate if the population of the Görtschitztal valley has experienced higher incidence of cancer in the study period 1983–2012. Cancer incidence including in situ and prostate cancer in St. Veit East was slightly above the average for Carinthia and St. Veit West in the majority of years studied. When separating by gender, male cancer incidence in St. Veit East was above average but female cancer incidence was average compared to the whole region of Carinthia while it was often higher compared to St. Veit West. When excluding prostate cancer and in situ cases from overall cancer incidence data, no overall increase in cancer incidence for Carinthia over the studied time period was observed and St. Veit East exhibited the same average cancer incidence as Carinthia overall.

Taking these observations together, the time trend of overall cancer incidence and the regional differences can be predominantly attributed to prostate cancer (and carcinoma in situ). Since the incidence of prostate cancer depends on participation in screening programs introducing a lead-time bias with earlier diagnoses of cancer, the time trend and the difference between St. Veit East and Carinthia may be due to awareness bias because in this region chemicals have been processed and disposed since the early twentieth century.

According to these analyses (Medical University of Vienna [7]) based on HCB measurements in spruce needles from the 3 preceding years, the period of elevated emission of HCB was up to 18 months starting in 2013. Therefore, any increase in cancer incidence rates in St. Veit East based on data until 2012 cannot be attributed to the HCB contamination of regional food products identified in 2014. While a contribution of the release of HCB into the environment can be excluded for the studied time period, the strongly increased incidence of mesotheliomas in St. Veit East could be explained by asbestos-related exposure as it is known that asbestos was processed until the end of the 1980s by the cement factory in this region. The time trend of mesothelioma incidence underlines the long latency periods for carcinomas, which are described in the scientific literature as being up to 30–40 years [8, 9]. Considering causal pathways, it is very unlikely that an effect of HCB exposure can be observed during the study period, because the increased exposure only started after mid-2013. Furthermore, long latency periods of cancer preclude an early impact on incidences even if there is an effect of HCB exposure at all. Until today, the latter has not been established by epidemiological studies [10].

It should be mentioned that at the beginning of the HCB crisis, monitoring programs for food were implemented as part of the risk management. These programs covered all types of foods produced in the proclaimed affected area, including drinking water, animal food, honey, vegetables and fruits, herbs, cereals, and vegetable oil. Furthermore, a human biomonitoring survey (blood samples) of the affected population was started 2015.

## Conclusion

Evaluation of cancer incidence in this report was confined to the period 1983–2012 for which reliable and nearly complete registry data are available. It is impossible that HCB had an impact on cancer incidence in the mentioned period up to 2012 because contamination did not start before 2013. This is in contrast to the newspaper report which was the starting point for the analyses presented here. The descriptive study did not show any remarkable overall differences in cancer incidence between Görtschitztal valley, district St. Veit/Glan excluding Görtschitztal valley and Carinthia excluding district St. Veit/Glan. In a sensitivity analysis carcinoma in situ cases as well as prostate cancer were excluded since completeness of case ascertainment is known to be dependent on participation in prostate screening programs and on the willingness of local physicians to report in situ cases [11]. Existing tumor-specific incident rate differences from the years prior to the HCB exposure suggest other environmental carcinogenic influences.

The results show that only a well-planned analysis and knowledge of pitfalls in dealing with registry data can provide an objective picture of the situation. This requires the interdisciplinary collaboration of (environmental) medical, epidemiological and statistical disciplines. Preliminary assessment of a complex medical situation without profound specific expertise carries the risk of inducing a biased attitude in the affected population, which may hardly be subsequently changed by facts and could lead to chronic psychological stress. Therefore, an overly simplified presentation of results via the media should be avoided in a situation where the population under risk is already feeling insecure by previous reports about massive health-related risks. News media representatives should be sensitized to the impact of oversimplified presentations of health data and encouraged to provide unbiased information to facilitate implementation of public health measures.
